# Some properties and applications of the Teodorescu operator associated to the Helmholtz equation

**DOI:** 10.1186/s13660-017-1537-2

**Published:** 2017-10-25

**Authors:** Pei Yang, Liping Wang, Long Gao

**Affiliations:** College of Mathematics and Information Science, Hebei Normal University, Shijiazhuang, Hebei 050024 P.R. China

**Keywords:** quaternion analysis, Teodorescu operator, Helmholtz equation, Riemann boundary value problem, integral representation

## Abstract

In this paper, we first define the Teodorescu operator $T_{\psi,\alpha }$ related to the Helmholtz equation and discuss its properties in quaternion analysis. Then we propose the Riemann boundary value problem related to the Helmholtz equation. Finally we give the integral representation of the boundary value problem by using the previously defined operator.

## Introduction

It is well known that the Helmholtz equation is an elliptic partial differential equation describing the electromagnetic wave, which has important applications in geophysics, medicine, engineering application, and many other fields. Many problems associated with the Helmholtz equation have been studied by many scholars, for example [1–5]. The boundary value problem for partial differential equations is an important and meaningful research topic. The singular integral operator is the core component of the solution of the boundary value problem for a partial differential system. The Teodorescu operator is a generalized solution of the inhomogeneous Dirac equation, which plays an important role in the integral representation of the general solution for the boundary value problem. Many experts and scholars have studied the properties of the Teodorescu operator. For example, Vekua [6] first discussed some properties of the Teodorescu operator on the plane and its application in thin shell theory and gas dynamics. Hile [7] and Gilbert [8] studied some properties of the Teodorescu operator in n-dimensional Euclid space and high complex space, respectively. Yang [9] and Gu [10] studied the boundary value problem associated with the Teodorescu operator in quaternion analysis and Clifford analysis, respectively. Wang [11–15] studied the properties of many Teodorescu operators and related boundary value problems.

In this paper, we will study some properties of the singular integral operator and the Riemann boundary value problem associated to the Helmholtz equation using the quaternion analysis method. The structure of this paper is as follows: in Section 2, we review some basic knowledge of quaternion analysis. In Section 3, we first construct a singular integral operator $T_{\psi,\alpha}$ related to the Helmholtz equation and study some of its properties. In Section 4, we propose the Riemann boundary value problem related to the Helmholtz equation. Finally we give the integral representation of the boundary value problem by using the previously defined operator.

## Preliminaries

Let $\{i_{1},i_{2},i_{3}\}$ be an orthogonal basis of the Euclidean space $R^{3}$ and $\mathbb{H}(\mathbb{C})$ be the set of complex quaternions with basis

$$ \{i_{0},i_{1},i_{2},i_{3}\}, $$

where $i_{0}$ is the unit and $i_{1}$, $i_{2}$, $i_{3}$ are the quaternionic imaginary units with the following properties:

$$\begin{aligned}& i_{0}^{2}=-i_{k}^{2}=i_{0}, \qquad i_{0}i_{k}=i_{k}i_{0}=i_{k}, \quad k=1,2,3, \\& i_{1}i_{2}=-i_{2}i_{1}=i_{3}, \qquad i_{2}i_{3}=-i_{3}i_{2}=i_{1}, \qquad i_{3}i_{1}=-i_{1}i_{3}=i_{2}. \end{aligned}$$

Then an arbitrary quaternion *a* can be written as $a=\sum_{k=0}^{3}a_{k}i_{k}$, $a_{k}\in\mathbb{C}$. The quaternionic conjugation is defined by $\bar{a}=a_{0}-\sum_{k=1}^{3}a_{k}\cdot i_{k}$. The norm for an element $a\in\mathbb{H}(\mathbb{C})$ is taken to be $|a|=\sqrt{\sum_{k=0}^{3}|a_{k}|^{2}}$. Moreover, if $a\bar{a}=\bar {a}a=|a|^{2}$ and $|a|\neq0$, then we say that *a* is reversible. Obviously, its inverse element can be written as $a^{-1}=\frac{\bar {a}}{|a|^{2}}$.

Let $\lambda\in\mathbb{C}\backslash\{0\}$ and let *α* be its complex square root: $\alpha\in\mathbb{C}$, $\alpha ^{2}=\lambda$. Suppose $\Omega\subset R^{3}$ is a domain and *∂*Ω is its boundary. We shall consider functions *f* defined in $\Omega\subset R^{3}$ with values in $\mathbb{H}(\mathbb {C})$. Then *f* can be expressed as $f=\sum_{k=0}^{3}f_{k}(x)i_{k}$. Here $f_{k}(x)$ ($k=0,1,2,3$) are complex functions defined on Ω.

Let $C^{(m)}(\Omega,\mathbb{H}(\mathbb{C}))=\{f\mid f:\Omega \rightarrow\mathbb{H}(\mathbb{C}), f(x)=\sum_{k=0}^{3}f_{k}(x)i_{k}, f_{k}(x)\in C^{m}(\Omega,\mathbb{C})\}$. We define the operators as follows:

$$\begin{aligned}& {{}^{\psi}D}[f] = \sum_{k=1}^{3} \psi_{k}\cdot\frac {\partial f}{\partial x_{k}},\qquad {{}^{\psi} \overline{D}}[f]=\sum_{k=1}^{3}\overline{ \psi}_{k}\cdot \frac{\partial f}{\partial x_{k}}, \\& {D^{\psi}[f]} = \sum_{k=1}^{3} \frac{\partial f}{\partial x_{k}}\cdot\psi_{k},\qquad \overline{D}^{\psi}[f]= \sum_{k=1}^{3}\frac{\partial f}{\partial x_{k}}\cdot \overline{\psi}_{k}, \end{aligned}$$

where $\psi=\{\psi_{1},\psi_{2},\psi_{3}\}=\{i_{1},i_{2},i_{3}\}$.

For the above stated *α*, let us introduce the following operators:

$$\begin{aligned}& {{}^{\psi}D}_{\alpha}[f]=\alpha f+{{}^{\psi}D}[f],\qquad {{}_{\alpha}D^{\psi}[f]=\alpha f+D^{\psi}[f]}, \\& {{}^{\psi}\overline{D}}_{\alpha}[f]=\alpha f-{{}^{\psi}D}[f], \qquad {{}_{\alpha}\overline{D}}^{\psi}[f]=\alpha f-D^{\psi}[f]. \end{aligned}$$

*f* will be called a left (right)-$(\psi,\alpha)$-hyperholomorphic in the domain Ω, if ${{}^{\psi}D}_{\alpha}[f]=0$ (${{}_{\alpha }D}^{\psi}[f]=0$) in Ω. Let $\alpha\in\mathbb{C}\backslash \{0\}$ and $\operatorname{Im}\alpha\neq0$. For $x\in R^{3}\backslash\{0\}$, we introduce the following notation:

$$\theta_{\alpha}(x)= \textstyle\begin{cases} -\frac{1}{4\pi|x|}e^{i\alpha|x|},&\operatorname{Im}\alpha>0, \\ -\frac{1}{4\pi|x|}e^{-i\alpha|x|},&\operatorname{Im}\alpha< 0. \end{cases} $$

In both cases it is a fundamental solution of the Helmholtz equation with $\lambda=\alpha^{2}$. Then the fundamental solution to the operator ${{}^{\psi}D}_{\alpha}$, $\mathcal{K}_{\psi,\alpha}$ is given by

$$\mathcal{K}_{\psi,\alpha}(x)={{}^{\psi}\overline{D}}_{\alpha }[ \theta_{\alpha}](x)= \textstyle\begin{cases} \theta_{\alpha}(x)(\alpha+\frac{x}{|x|^{2}}-i\alpha \frac{x}{|x|}),&\operatorname{Im}\alpha>0, \\ \theta_{\alpha}(x)(\alpha+\frac{x}{|x|^{2}}+i\alpha \frac{x}{|x|}),&\operatorname{Im}\alpha< 0. \end{cases} $$

If $f(x)\in L^{p,\sigma}(R^{3},\mathbb{H}(\mathbb{C}))$ means that $f(x)\in L^{p}(B,\mathbb{H}(\mathbb{C}))$, $f^{(\sigma )}(x)=|x|^{-\sigma}f(\frac{\overline{x}}{|x|^{2}})\in L^{p}(B,\mathbb{H}(\mathbb{C}))$, in which $B=\{x\mid |x|<1\}$, *σ* is a real number, $\|f\|_{p,\sigma}=\|f\|_{L^{p}(B)}+\|f^{(\sigma )}\|_{L^{p}(B)}$, $p\geq1$.

### Definition 2.1

Suppose that the functions *u*, *v*, *φ* are defined in Ω with values in $\mathbb{H}(\mathbb{C})$ and $u, v\in L^{1}(\Omega,\mathbb{H}(\mathbb{C}))$. If, for arbitrary $\varphi\in C_{0}^{\infty}(\Omega,\mathbb{H}(\mathbb{C}))$, *u*, *v* satisfy

$$\int_{\Omega}\varphi(x)u(x)\,dv_{x}- \int_{\Omega}{ _{\alpha}\overline{D}^{\psi}[ \varphi]v(x)\,dv_{x}}=0, $$

then *u* is called a generalized derivative of the function *v*, where we denote $u={{}^{\psi}D}_{\alpha}[v]$.

### Lemma 2.1

([16])

*If* $\sigma_{1},\sigma_{2}>0$, $0\leq \gamma\leq1$, *then we have*

$$\bigl\vert \sigma_{1}^{\gamma}-\sigma_{2}^{\gamma} \bigr\vert \leq \vert \sigma_{1}-\sigma _{2} \vert ^{\gamma}. $$

### Lemma 2.2

([17])

*Suppose* Ω *is a bounded domain in* $R^{3}$ *and let* $\alpha'$, $\beta'$ *satisfy* $0<\alpha', \beta'<3$, $\alpha'+\beta'>3$. *Then*, *for all* $x_{1},x_{2}\in R^{3}$ *and* $x_{1}\neq x_{2}$, *we have*

$$\int_{\Omega} \vert t-x_{1} \vert ^{-\alpha'} \vert t-x_{2} \vert ^{-\beta '}\,dt\leq M_{0}\bigl( \alpha',\beta'\bigr) \vert x_{1}-x_{2} \vert ^{3-\alpha'-\beta'}. $$

### Lemma 2.3

([18])

*Let* Ω, *∂*Ω *be as stated above*. *If* $f \in C^{(m)}(\overline{\Omega},\mathbb{H}(\mathbb {C}))$ ($m\geq1$), *then we have*

$$\int_{\partial\Omega}f(y)\,d\sigma_{y}\mathcal {K}_{\psi,\alpha}(y-x)+ \int_{\Omega} {{}_{\alpha}\overline{D}^{\psi } \bigl[f(y)\bigr]\mathcal{K}_{\psi,\alpha}(y-x)\,dv_{y}}=f(x),\quad x \in \Omega. $$

## Some properties of the singular integral operator $T_{\psi ,\alpha}$ for the Helmholtz equation

In this section, we will discuss some properties of the singular integral operators as follows:

3.1 $$\begin{aligned}& \bigl(T_{\psi,\alpha}[f]\bigr) (x) \\& \quad = \int_{B}\mathcal{K}_{\psi,\alpha}(y-x)f(y) \,dv_{y}+ \int_{B}\mathcal{K}_{\psi,\alpha}\biggl(\frac{\overline {y}}{|y|^{2}}-x \biggr)f \biggl(\frac{\overline{y}}{|y|^{2}} \biggr)\frac {1}{|y|^{6}}\,dv_{y} \\& \quad = \bigl(T_{\psi,\alpha}^{(1)}[f]\bigr) (x)+\bigl(T_{\psi,\alpha }^{(2)}[f] \bigr) (x), \end{aligned}$$

where $B=\{x\mid |x|<1\}$, $\alpha=a+ib$, $b>0$.

### Theorem 3.1

*Assume* *B* *to be as stated above*, $\alpha=a+ib$, $b>0$. *If* $f\in L^{p}(B,\mathbb{H}(\mathbb{C}))$, $3< p<+\infty$, *then*

1. $|(T_{\psi,\alpha}^{(1)}[f])(x)|\leq M_{1}(p)\|f\| _{L^{p}(B)}$, $x\in R^{3}$,
2. $|(T_{\psi,\alpha}^{(1)}[f])(x_{1})-(T_{\psi ,\alpha}^{(1)}[f])(x_{2})|\leq M_{2}(p)\|f\|_{L^{p}(B)}|x_{ 1}-x_{2}|+ M_{3}(p)\|f\|_{L^{p}(B)}|x_{1}-x_{2}|^{\beta}$, $x_{ 1}, x_{2}\in R^{3}$,
3. ${{}^{\psi}D}_{\alpha}(T_{\psi,\alpha }^{(1)}[f])(x)=f(x)$, $x\in B$, ${{}^{\psi}D}_{\alpha}(T_{\psi,\alpha }^{(1)}[f])(x)=0$, $x\in R^{3}\backslash\overline{B}$,

*where* $0<\beta=\frac{p-3}{p}<1$.

### Proof

(1)

$$\begin{aligned} \bigl(T_{\psi,\alpha}^{(1)}[f]\bigr) (x) =& \int _{B}\mathcal{K}_{\psi,\alpha}(y-x)f(y) \,dv_{y} \\ =& -\frac{\alpha}{4\pi} \int_{B}\frac {e^{i\alpha|y-x|}}{|y-x|}f(y)\,dv_{y} - \frac{1}{4\pi} \int_{B}\frac{e^{i\alpha|y- x|}(y-x)}{|y-x|^{3}}f(y)\,dv_{y} \\ &{}+ \frac{i\alpha}{4\pi} \int_{B}\frac{e^{i\alpha|y -x|}(y-x)}{|y-x|^{2}}f(y)\,dv_{y} \\ =& I_{1}+I_{2}+I_{3}. \end{aligned}$$

(i) By the Taylor series, we have $|e^{i\alpha |y-x|}|=|e^{i(a+ib)|y-x|}|=e^{-b|y-x|}\leq\frac{1}{b|y-x|}$. By the Hölder inequality, we have

3.2 $$\begin{aligned} |I_{1}| \leq& \frac{|\alpha|}{4\pi} \int_{B}\frac {e^{-b|y-x|}}{|y-x|} \bigl\vert f(y) \bigr\vert \,dv_{y}\leq J_{1} \int_{B}\frac {1}{|y-x|^{2}} \bigl\vert f(y) \bigr\vert \,dv_{y} \\ \leq& J_{1}\|f\|_{L^{p}(B)} \biggl[ \int_{B}\frac {1}{|y-x|^{2q}}\,dv_{y} \biggr]^{\frac{1}{q}}. \end{aligned}$$

When $x\in\overline{B}$, because $p>3$, $\frac{1}{p}+\frac {1}{q}=1$. Then $1< q<\frac{3}{2}$. Thus $\int_{B}\frac{1}{|y-x|^{2q}}\,dv_{y}$ is bounded. Hence we suppose

3.3 $$ \int_{B}\frac{1}{|y-x|^{2q}}\,dv_{y}\leq J_{2}. $$

When $x\in R^{3}\backslash\overline{B}$, by Lemma 2.1 and the generalized spherical coordinate, we have

3.4 $$ \int_{B}\frac{1}{|y-x|^{2q}}\,dv_{y}\leq J_{3} \int _{d_{0}}^{d_{0}+2}\rho^{2-2q}\,d\rho\leq J_{4}, $$

where $\rho=|y-x|$, $d_{0}=d(x,B)$. Therefore, for arbitrary $x\in R^{3}$, we obtain

3.5 $$ |I_{1}|\leq M_{1}^{(1)}(p)\|f\|_{L^{p}(B)}, $$

where $M_{1}^{(1)}(p)=\max\{J_{1}J_{2}^{\frac {1}{q}},J_{1}J_{4}^{\frac{1}{q}}\}$.

(ii) Obviously, $e^{-b|y-x|}\leq1$. By the Hölder inequality, we have

$$\begin{aligned} |I_{2}| \leq& \frac{1}{4\pi} \int_{B}\frac {e^{-b|y-x|}}{|y-x|^{2}} \bigl\vert f(y) \bigr\vert \,dv_{y}\leq J_{5} \int _{B}\frac{1}{|y-x|^{2}} \bigl\vert f(y) \bigr\vert \,dv_{y} \\ \leq& J_{5}\|f\|_{L^{p}(B)} \biggl[ \int_{B}\frac {1}{|y-x|^{2q}}\,dv_{y} \biggr]^{\frac{1}{q}}. \end{aligned}$$

Then, by inequality (3.3) and (3.4), we have

3.6 $$ |I_{2}|\leq M_{1}^{(2)}(p)\|f\|_{L^{p}(B)}, $$

where $M_{1}^{(2)}(p)=\max\{J_{5}J_{2}^{\frac {1}{q}},J_{5}J_{4}^{\frac{1}{q}}\}$.

(iii) This case is similar to (ii). We obtain

3.7 $$ |I_{3}|\leq M_{1}^{(3)}(p)\|f\|_{L^{p}(B)}. $$

By inequalities (3.5)-(3.7), we obtain

$$ \bigl\vert \bigl(T_{\psi,\alpha}^{(1)}[f]\bigr) (x) \bigr\vert \leq |I_{1}|+|I_{2}|+|I_{3}|\leq M_{1}(p)\|f\|_{L^{p}(B)}, $$

where $M_{1}(p)=M_{1}^{(1)}(p)+M_{1}^{(2)}(p)+M_{1}^{(3)}(p)$.

$$\begin{aligned}& (2)\quad \bigl(T_{\psi,\alpha}^{(1)}[f]\bigr) (x_{1})- \bigl(T_{\psi ,\alpha}^{(1)}[f]\bigr) (x_{2}) \\& \hphantom{(2)\quad}\quad = \int_{B}\bigl[\mathcal{K}_{\psi ,\alpha}(y-x_{1})- \mathcal{K}_{\psi,\alpha}(y- x_{2})\bigr]f(y)\,dv_{y} \\& \hphantom{(2)\quad}\quad = -\frac{\alpha}{4\pi} \int_{B} \biggl[ \frac{e^{i\alpha|y-x_{1}|}}{|y-x_{1}|}-\frac {e^{i\alpha|y-x_{2}|}}{|y-x_{2}|} \biggr]f(y)\,dv_{ y} \\& \hphantom{(2)\quad}\qquad {}-\frac{1}{4\pi} \int_{B} \biggl[\frac{e^{i\alpha |y-x_{1}|}(y-x_{1})}{|y-x_{1}|^{3}}-\frac{e^{ i\alpha|y-x_{2}|}(y-x_{2})}{|y-x_{2}|^{3}} \biggr] f(y)\,dv_{y} \\& \hphantom{(2)\quad}\qquad {} +\frac{i\alpha}{4\pi} \int_{B} \biggl[ \frac{e^{i\alpha|y-x_{1}|}(y-x_{1})}{|y-x_{1}|^{2}} -\frac{e^{i\alpha|y-x_{2}|}(y-x_{2})}{|y- x_{2}|^{2}} \biggr]f(y)\,dv_{y} \\& \hphantom{(2)\quad}\quad = I_{4}+I_{5}+I_{6}. \end{aligned}$$

Let us consider $e^{i\alpha|y-x|}$. For arbitrary $x\in R^{3}$, it is easy to prove $|e^{i\alpha|y-x|}|\leq1$ and satisfy $|e^{i\alpha|y-x_{1}|}-e^{i\alpha|y-x_{2}|}|\leq c|x_{1}-x_{2}|$.

(i) For arbitrary $x_{1}, x_{2}\in R^{3}$, by the Hölder inequality, we have

$$\begin{aligned} |I_{4}| \leq& J_{6} \int_{B} \biggl\vert \frac {e^{i\alpha|y-x_{1}|}}{|y-x_{1}|}-\frac{e^{i\alpha |y-x_{2}|}}{|y-x_{2}|} \biggr\vert \bigl\vert f(y) \bigr\vert \,dv_{y} \\ \leq& J_{6} \int_{B}\frac{|e^{i\alpha|y -x_{1}|}-e^{i\alpha|y-x_{2}|}|}{|y-x_{1}|} \bigl\vert f(y) \bigr\vert \,dv_{y} + J_{6} \int_{B} \biggl\vert e^{i\alpha|y-x_{2}|} \biggl( \frac {1}{|y-x_{1}|}-\frac{1}{|y-x_{2}|} \biggr) \biggr\vert \bigl\vert f(y) \bigr\vert \,dv_{y} \\ \leq& J_{7} \int_{B}\frac{1}{|y- x_{1}|} \bigl\vert f(y) \bigr\vert \,dv_{y}|x_{1}-x_{2}| +J_{6} \int_{B}\frac{1}{|y-x_{1}||y- x_{2}|} \bigl\vert f(y) \bigr\vert \,dv_{y}|x_{1}-x_{2}| \\ \leq& \biggl\{ J_{7} \biggl\{ \int_{B}\frac{1}{|y- x_{1}|^{q}}\,dv_{y} \biggr\} ^{\frac{1}{q}}+ J_{6} \biggl\{ \int_{B}\frac{1}{|y-x_{1}|^{q}|y- x_{2}|^{q}}\,dv_{y} \biggr\} ^{\frac{1}{q}} \biggr\} \|f\|_{L^{p}( B)}|x_{1}-x_{2}|. \end{aligned}$$

As $1< q<\frac{3}{2}$, $\int_{B}\frac {1}{|y-x_{1}|^{q}|y-x_{2}|^{q}}\,dv_{y}$ and $\int_{B}\frac {1}{|y-x_{1}|^{q}}\,dv_{y}$ are bounded. Hence

3.8 $$\begin{aligned}& |I_{4}|\leq M_{2}^{(1)}(p)\|f\|_{L^{p}(B)}|x_{1}-x_{2}|. \\& (\mathrm{ii})\quad I_{5}= -\frac{1}{4\pi} \int_{B} \biggl[\frac {e^{i\alpha|y-x_{1}|}(y-x_{1})}{|y-x_{1}|^{3}}-\frac{e^{i\alpha |y-x_{2}|}(y-x_{2})}{|y-x_{2}|^{3}} \biggr]f(y)\,dv_{y} \\& \hphantom{(\mathrm{ii})\quad I_{5}}= -\frac{1}{4\pi} \int_{B}\frac{(e^{i\alpha|y -x_{1}|}-e^{i\alpha|y-x_{2}|})(y- x_{1})}{|y-x_{1}|^{3}}f(y)\,dv_{y} \\& \hphantom{(\mathrm{ii})\quad I_{5}={}}{}- \frac{1}{4\pi} \int_{B}e^{i\alpha|y- x_{2}|} \biggl(\frac{y-x_{1}}{|y-x_{1}|^{3}}- \frac{y-x_{2}}{|y-x_{2}|^{3}} \biggr)f(y)\,dv_{y} \\& \hphantom{(\mathrm{ii})\quad I_{5}}=I_{5}^{(1)}+I_{5}^{(2)}. \end{aligned}$$

By the Hölder inequality, we have

$$\begin{aligned} \bigl\vert I_{5}^{(1)} \bigr\vert \leq& \frac{1}{4\pi} \int_{B}\frac {|e^{i\alpha|y-x_{1}|}-e^{i\alpha|y-x_{2}|}|}{|y- x_{1}|^{2}} \bigl\vert f(y) \bigr\vert \,dv_{y} \\ \leq& J_{8} \int_{B}\frac{1}{|y- x_{1}|^{2}} \bigl\vert f(y) \bigr\vert \,dv_{y}|x_{1}-x_{2}| \\ \leq& J_{8}\|f\|_{L^{p}(B)} \biggl[ \int_{B}\frac {1}{|y-x_{1}|^{2q}}\,dv_{y} \biggr]^{\frac{1}{q}}|x_{1}-x_{2}|. \end{aligned}$$

As $1< q<\frac{3}{2}$, $\int_{B}\frac{1}{|y-x_{1}|^{2q}}\,dv_{y}$ is bounded. So we have

3.9 $$ \bigl\vert I_{5}^{(1)} \bigr\vert \leq J_{9} \|f\|_{L^{p}(B)}|x_{1}-x_{2}|. $$

By the Hölder inequality and the Hile lemma, we have

$$\begin{aligned} \bigl\vert I_{5}^{(2)} \bigr\vert \leq& \frac{1}{4\pi} \int_{B}\bigl|e^{i\alpha |y-x_{2}|}\bigr| \biggl\vert \frac{y-x_{1}}{|y-x_{1}|^{3}}- \frac {y-x_{2}}{|y-x_{2}|^{3}} \biggr\vert \bigl\vert f(y) \bigr\vert \,dv_{y} \\ \leq& J_{10} \int_{B} \biggl\vert \frac{y- x_{1}}{|y-x_{1}|^{3}}-\frac{y-x_{2}}{|y- x_{2}|^{3}} \biggr\vert \bigl\vert f(y) \bigr\vert \,dv_{y} \\ \leq& J_{10} \int_{B}\frac{|y-x_{1}|+|y- x_{2}|}{|y-x_{1}|^{2}|y-x_{2}|^{2}}|x_{1}-x_{2}| \bigl\vert f(y) \bigr\vert \,dv_{ y} \\ =& J_{10} \biggl\{ \int_{B}\frac{1}{|y- x_{1}||y-x_{2}|^{2}} \bigl\vert f(y) \bigr\vert \,dv_{y}+ \int_{B}\frac{1}{|y- x_{1}|^{2}|y-x_{2}|} \bigl\vert f(y) \bigr\vert \,dv_{y} \biggr\} |x_{1}-x_{2}| \\ \leq& J_{10} \biggl\{ \biggl[ \int_{B}\frac {1}{|y-x_{1}|^{q}|y-x_{2}|^{2q}}\,dv_{y} \biggr] ^{\frac{1}{q}} + \biggl[ \int_{B}\frac{1}{|y-x_{1}|^{2q}|y-x_{ 2}|^{q}}\,dv_{y} \biggr]^{\frac{1}{q}} \biggr\} \\ &{}\times\|f\|_{ L^{p}(B)}|x_{1}-x_{2}|. \end{aligned}$$

We suppose $\alpha'=q$, $\beta'=2q$. As $1< q<\frac{3}{2}$, we have $\alpha'=q<3$, $\beta'=2q<3$, $\alpha'+\beta'=3q>3$. Hence, by Lemma 2.2, we have

$$\begin{aligned}& \int_{B}\frac{1}{|y-x_{1}|^{q}|y-x_{2}|^{2q}}\,dv_{y}\leq M_{0}\bigl(\alpha',\beta' \bigr)|x_{1}-x_{2}|^{3-3q}, \\& \int_{B}\frac{1}{|y-x_{1}|^{2q}|y-x_{2}|^{q}}\,dv_{y}\leq M_{0}\bigl(\alpha',\beta' \bigr)|x_{1}-x_{2}|^{3-3q}. \end{aligned}$$

So we have

3.10 $$ \bigl\vert I_{5}^{(2)} \bigr\vert \leq J_{11} \|f\| _{L^{p}(B)}\bigl(|x_{1}-x_{2}|^{3-3q} \bigr)^{\frac{1}{q}}|x_{1}-x_{2}| =J_{11}\|f \|_{L^{p}(B)}|x_{1}-x_{2}|^{\beta}, $$

where $0<\beta=\frac{p-3}{p}<1$. By inequality (3.9) and (3.10), we have

3.11 $$\begin{aligned}& |I_{5}|\leq J_{9}\|f\|_{L^{p}(B)}|x_{1}-x_{2}|+J_{11} \|f\| _{L^{p}(B)}|x_{1}-x_{2}|^{\beta}. \\& (\mathrm{iii})\quad I_{6}= \frac{i\alpha}{4\pi} \int_{B} \biggl[\frac{e^{i\alpha|y-x_{1}|}(y-x_{1})}{|y-x_{1}|^{2}}-\frac {e^{i\alpha|y-x_{2}|}(y-x_{2})}{|y-x_{2}|^{2}} \biggr]f(y)\,dv_{y} \\& \hphantom{(\mathrm{iii})\quad I_{6}}= \frac{i\alpha}{4\pi} \int_{B}\frac{(e^{i\alpha |y-x_{1}|}-e^{i\alpha|y-x_{2}|})(y-x_{1})}{|y-x_{1}|^{2}}f(y)\,dv_{y} \\& \hphantom{(\mathrm{iii})\quad I_{6}={}}{}+ \frac{i\alpha}{4\pi} \int_{B}e^{i\alpha |y-x_{2}|} \biggl(\frac{y-x_{1}}{|y-x_{1}|^{2}}- \frac {y-x_{2}}{|y-x_{2}|^{2}} \biggr)f(y)\,dv_{y} \\& \hphantom{(\mathrm{iii})\quad I_{6}}=I_{6}^{(1)}+I_{6}^{(2)}. \end{aligned}$$

Similar to $I_{5}^{(1)}$, we have

3.12 $$ \bigl\vert I_{6}^{(1)} \bigr\vert \leq J_{12} \|f\|_{L^{p}(B)}|x_{1}-x_{2}|. $$

By the Hölder inequality and the Hile lemma, we have

$$\begin{aligned} \bigl\vert I_{6}^{(2)} \bigr\vert \leq& \frac{|\alpha|}{4\pi} \int_{B}\bigl|e^{i\alpha |y-x_{2}|}\bigr| \biggl\vert \frac{y-x_{1}}{|y-x_{1}|^{2}}- \frac {y-x_{2}}{|y-x_{2}|^{2}} \biggr\vert \bigl\vert f(y) \bigr\vert \,dv_{y} \\ \leq& J_{13} \int_{B} \biggl\vert \frac {y-x_{1}}{|y-x_{1}|^{2}}-\frac{y-x_{2}}{|y-x_{2}|^{2}} \biggr\vert \bigl\vert f(y) \bigr\vert \,dv_{y} \\ \leq& J_{13} \int_{B}\frac {|x_{1}-x_{2}|}{|y-x_{1}||y-x_{2}|} \bigl\vert f(y) \bigr\vert \,dv_{y} \\ =& J_{13} \int_{B}\frac {1}{|y-x_{1}||y-x_{2}|} \bigl\vert f(y) \bigr\vert \,dv_{y}|x_{1}-x_{2}| \\ \leq& J_{13}\|f\|_{L^{p}(B)} \biggl\{ \int_{B}\frac {1}{|y-x_{1}|^{q}|y-x_{2}|^{q}}\,dv_{y} \biggr\} ^{\frac{1}{q}}|x_{1}-x_{2}|. \end{aligned}$$

As $1< q<\frac{3}{2}$, $\int_{B}\frac {1}{|y-x_{1}|^{q}|y-x_{2}|^{q}}\,dv_{y}$ is bounded. So we have

3.13 $$ \bigl\vert I_{6}^{(2)} \bigr\vert \leq J_{14} \|f\|_{L^{p}(B)}|x_{1}-x_{2}|. $$

By inequalities (3.12) and (3.13), we have

3.14 $$ |I_{6}|\leq \bigl\vert I_{6}^{(1)} \bigr\vert + \bigl\vert I_{6}^{(2)} \bigr\vert \leq M_{2}^{(2)}(p)\|f\| _{L^{p}(B)}|x_{1}-x_{2}|, $$

where $M_{2}^{(2)}(p)=J_{12}+J_{14}$. By inequalities (3.8), (3.11) and (3.14), we have

$$\bigl\vert \bigl(T_{\psi,\alpha}^{(1)}[f]\bigr) (x_{1})- \bigl(T_{\psi,\alpha }^{(1)}[f]\bigr) (x_{2}) \bigr\vert \leq M_{2}(p)\|f\|_{L^{p}(B)}|x_{1}-x_{2}|+ M_{3}(p)\|f\|_{L^{p}(B)}|x_{1}-x_{2}|^{\beta}, $$

where $M_{2}(p)=M_{2}^{(1)}(p)+J_{9}+M_{2}^{(2) }(p)$, $M_{3}(p)=J_{11}$.

(3) When $x\in B$, for arbitrary $\varphi\in C_{0}^{\infty}(B,\mathbb{H}(\mathbb{C}))$, by Lemma 2.3 and the Fubini theorem, we have

$$\begin{aligned} \int_{B}{{}_{\alpha}\overline{D}}^{\psi}[\varphi ]\bigl(T_{\psi,\alpha}^{(1)}[f]\bigr) (x)\,dv_{x} =& \int_{B}{{}_{\alpha}\overline{D}}^{\psi}[\varphi ] \biggl[ \int_{B}\mathcal{K}_{\psi,\alpha}(y-x)f(y)\,dv_{y} \biggr]\,dv_{x} \\ =& \int_{B} \biggl[ \int_{B}{{}_{\alpha}\overline {D}}^{\psi}[ \varphi]\mathcal{K}_{\psi,\alpha}(y-x)\,dv_{x} \biggr]f(y) \,dv_{y} \\ =& \int_{B} \biggl[\varphi(y)- \int_{\partial B}\varphi (x)\,d\sigma_{x} \mathcal{K}_{\psi,\alpha}(y-x) \biggr]f(y)\,dv_{y} \\ =& \int_{B}\varphi(y)f(y)\,dv_{y}= \int_{B}\varphi(x)f(x)\,dv_{x}. \end{aligned}$$

Hence, in the sense of generalized derivatives, ${{}^{\psi}D}_{\alpha }(T_{\psi,\alpha}^{(1)}[f])(x)=f(x)$, $x\in B$. When $x\in R^{3}\backslash\overline{B}$, it is easy to see ${{}^{\psi}D}_{\alpha }(T_{\psi,\alpha}^{(1)}[f])(x)=0$. □

### Theorem 3.2

*Assume* *B* *to be as stated above and* $\alpha=a+ib$, $b>0$.*If* $f\in L^{p,3}(B,\mathbb{H}(\mathbb{C}))$, $3< p<+\infty$, *then*

1. $|(T_{\psi,\alpha}^{(2)}[f])(x)|\leq M_{4}(p)\| f^{(3)}\|_{L^{p}(B)}$, $x\in R^{3}$,
2. $|(T_{\psi,\alpha}^{(2)}[f])(x_{1})-(T_{ \psi,\alpha}^{(2)}[f])(x_{2})|\leq M_{5}(p)\|f^{(3)}\|_{ L^{p}(B)}|x_{1}-x_{2}|+ M_{6}(p)\|f^{(3)}\|_{L^{p}(B)}|x_{1}-x_{2}|^{\beta }$, $x_{1}, x_{2}\in R^{3}$,
3. ${{}^{\psi}D}_{\alpha}(T_{\psi,\alpha }^{(2)}[f])(x)=0$, $x\in B$, ${{}^{\psi}D}_{\alpha}(T_{\psi,\alpha }^{(2)}[f])(x)=f(x)$, $x\in R^{3}\backslash\overline{B}$,

*where* $0<\beta=\frac{p-3}{p}<1$.

### Proof

(1)

$$\begin{aligned} \bigl(T_{\psi,\alpha}^{(2)}[f]\bigr) (x) =& \int_{B}\mathcal{K}_{\psi,\alpha} \biggl(\frac{\overline {y}}{|y|^{2}}-x \biggr)f \biggl(\frac{\overline{y}}{|y|^{2}} \biggr)\frac{1}{|y|^{6}}\,dv_{y} \\ =& -\frac{\alpha}{4\pi} \int_{B}\frac{e^{i\alpha |\frac{\overline{y}}{|y|^{2}}-x|}}{|\frac{\overline {y}}{|y|^{2}}-x|}f \biggl(\frac{\overline{y}}{|y|^{2}} \biggr) \frac {1}{|y|^{6}}\,dv_{y} \\ &{} -\frac{1}{4\pi} \int_{B}\frac{e^{i\alpha|\frac {\overline{y}}{|y|^{2}}-x|}(\frac{\overline{y}}{|y|^{2}}-x)}{|\frac {\overline{y}}{|y|^{2}}-x|^{3}} f \biggl(\frac{\overline {y}}{|y|^{2}} \biggr) \frac{1}{|y|^{6}}\,dv_{y} \\ &{}+ \frac{i\alpha}{4\pi} \int_{B} \frac{e^{i\alpha |\frac{\overline{y}}{|y|^{2}}-x|}(\frac{\overline {y}}{|y|^{2}}-x)}{|\frac{\overline{y}}{|y|^{2}}-x|^{2}}f \biggl(\frac {\overline{y}}{|y|^{2}} \biggr) \frac{1}{|y|^{6}}\,dv_{y} \\ =&I_{7}+I_{8}+I_{9}. \end{aligned}$$

As the first step, by the Hölder inequality, we have

3.15 $$\begin{aligned} |I_{7}| \leq& \frac{|\alpha|}{4\pi} \int_{B}\frac {e^{-b|\frac{\overline{y}}{|y|^{2}}-x|}}{|\frac{\overline {y}}{|y|^{2}}-x|} \biggl\vert f \biggl( \frac{\overline {y}}{|y|^{2}} \biggr) \biggr\vert \frac{1}{|y|^{6}}\,dv_{y} \\ \leq& C_{1} \int_{B}\frac{1}{|\frac{\overline {y}}{|y|^{2}}-x|} \biggl\vert f \biggl( \frac{\overline {y}}{|y|^{2}} \biggr) \biggr\vert \frac{1}{|y|^{6}}\,dv_{y} \\ \leq& C_{1} \biggl\{ \int_{B} \biggl[|y|^{-3} \biggl\vert f \biggl( \frac{\overline{y}}{|y|^{2}} \biggr) \biggr\vert \biggr]^{p}\,dv_{y} \biggr\} ^{\frac{1}{p}} \biggl\{ \int_{B}\frac {1}{|\frac{\overline{y}}{|y|^{2}}-x|^{q}|y|^{3q}}\,dv_{y} \biggr\} ^{\frac{1}{q}} \\ =&C_{1} \bigl\Vert f^{(3)} \bigr\Vert _{L^{p}(B)} \bigl[O_{1}(x) \bigr]^{\frac{1}{q}}, \end{aligned}$$

where $\frac{1}{p}+\frac{1}{q}=1$. Next we discuss $O_{1}(x)$ in two cases.

(i) When $|x|\geq\frac{1}{2}$, since

$$\begin{aligned} \biggl\vert \frac{\overline{y}}{|y|^{2}}-x \biggr\vert ^{-q}|y|^{-3q} =& |y|^{-2q} \biggl\{ |y|^{-q} \biggl\vert \frac{\overline {y}}{|y|^{2}}-x \biggr\vert ^{-q} \biggl\vert \frac{\overline{x}}{|x|^{2}} \biggr\vert ^{-q} \biggr\} |x|^{-q} \\ \leq& C_{2}|y|^{-2q} \biggl\vert y \biggl( \frac{\overline {y}}{|y|^{2}}-x \biggr)\frac{\overline{x}}{|x|^{2}} \biggr\vert ^{-q}|x|^{-q}= C_{2}|y|^{-2q} \biggl\vert \frac{\overline {x}}{|x|^{2}}-y \biggr\vert ^{-q}|x|^{-q}, \end{aligned}$$

we have

$$O_{1}(x)\leq \int_{B}C_{2}|y|^{-2q} \biggl\vert \frac {\overline{x}}{|x|^{2}}-y \biggr\vert ^{-q}|x|^{-q} \,dv_{y}=C_{2}|x|^{-q} \int _{B}|y|^{-2q} \biggl\vert \frac{\overline{x}}{|x|^{2}}-y \biggr\vert ^{-q}\,dv_{y}. $$

We suppose $\alpha'=2q$, $\beta'=q$. As $1< q<\frac{3}{2}$, we have $0<\alpha'<3$, $0<\beta'<3$, $\alpha'+\beta'=3q>3$. Thus, by Lemma 2.2, we have

3.16 $$ O_{1}(x)\leq C_{2}M_{0}\bigl( \alpha',\beta'\bigr)|x|^{-q} \biggl\vert \frac{\overline{x}}{|x|^{2}} \biggr\vert ^{3-3q}\leq C_{2}M_{0} \bigl(\alpha ',\beta'\bigr)2^{3-2q}= C_{3}. $$

(ii) When $|x|<\frac{1}{2}$, by $|y|<1$, we have $|1-yx|\geq \frac{1}{2}$, thus

3.17 $$\begin{aligned} O_{1}(x) =& \int_{B}\frac{1}{|\frac{\overline {y}}{|y|^{2}}-x|^{q}|y|^{3q}}\,dv_{y}= \int _{B}|y|^{-2q}|y|^{-q} \biggl\vert \frac{\overline{y}}{|y|^{2}}-x \biggr\vert ^{-q}\,dv_{y} \\ \leq& C_{4} \int_{B}|y|^{-2q} \biggl\vert y \biggl( \frac {\overline{y}}{|y|^{2}}-x \biggr) \biggr\vert ^{-q}\,dv_{y}= C_{4} \int_{B}|y|^{-2q}|1-yx|^{-q} \,dv_{y} \\ \leq& C_{4} \int_{B}|y|^{-2q}2^{q}\,dv_{y} \leq C_{5} \int_{B}|y|^{-2q}\,dv_{y}\leq C_{6}. \end{aligned}$$

Therefore, by (3.15)-(3.17), we have

3.18 $$ |I_{7}|\leq M_{4}^{(1)}(p) \bigl\Vert f^{(3)} \bigr\Vert _{L^{p}(B)}, $$

where $M_{4}^{(1)}(p)=\max\{C_{1}C_{3}^{\frac {1}{q}},C_{1}C_{6}^{\frac{1}{q}}\}$.

As the second step, by the Hölder inequality, we have

3.19 $$\begin{aligned} |I_{8}| \leq& \frac{1}{4\pi} \int_{B}\frac {e^{-b|\frac{\overline{y}}{|y|^{2}}-x|}}{|\frac{\overline {y}}{|y|^{2}}-x|^{2}} \biggl\vert f \biggl( \frac{\overline {y}}{|y|^{2}} \biggr) \biggr\vert \frac{1}{|y|^{6}}\,dv_{y} \\ \leq& C_{7} \int_{B}\frac{1}{|\frac{\overline {y}}{|y|^{2}}-x|^{2}} \biggl\vert f \biggl( \frac{\overline {y}}{|y|^{2}} \biggr) \biggr\vert \frac{1}{|y|^{6}}\,dv_{y} \\ \leq& C_{7} \biggl\{ \int_{B} \biggl[|y|^{-3} \biggl\vert f \biggl( \frac{\overline{y}}{|y|^{2}} \biggr) \biggr\vert \biggr]^{p}\,dv_{y} \biggr\} ^{\frac{1}{p}} \biggl\{ \int_{B}\frac{1}{|\frac {\overline{y}}{|y|^{2}}-x|^{2q}|y|^{3q}}\,dv_{y} \biggr\} ^{\frac {1}{q}} \\ =&C_{7}\bigl\| f^{(3)}\bigr\| _{L^{p}(B)} \bigl[O_{2}(x) \bigr]^{\frac{1}{q}}. \end{aligned}$$

Similar to $O_{1}(x)$, we find that $O_{2}(x)$ is bounded. Suppose $O_{2}(x)\leq C_{8}$. Then

3.20 $$ |I_{8}|\leq M_{4}^{(2)}(p) \bigl\Vert f^{(3)} \bigr\Vert _{L^{p}(B)}. $$

As the third step, similar to $I_{7}$, we have

3.21 $$ |I_{9}|\leq M_{4}^{(3)}(p) \bigl\Vert f^{(3)} \bigr\Vert _{L^{p}(B)}. $$

By inequalities (3.18), (3.20), and (3.21),

$$\bigl\vert \bigl(T_{\psi,\alpha}^{(2)}[f]\bigr) (x) \bigr\vert \leq|I_{7}|+|I_{8}|+|I_{9}|\leq M_{4}(p) \bigl\Vert f^{(3)} \bigr\Vert _{L^{p}(B)}, $$

where $M_{4}(p)=M_{4}^{(1)}(p)+M_{4}^{(2)}(p)+M_{4}^{(3) }(p)$.

$$\begin{aligned}& (2)\quad \bigl(T_{\psi,\alpha}^{(2)}[f]\bigr) (x_{1})- \bigl(T_{\psi,\alpha }^{(2)}[f]\bigr) (x_{2}) \\& \hphantom{(2)\quad}\quad = -\frac{\alpha}{4\pi} \int_{B} \biggl[\frac {e^{i\alpha|\frac{\overline{y}}{|y|^{2}}-x_{1}|}}{|\frac {\overline{y}}{|y|^{2}}-x_{1}|}-\frac{e^{i\alpha|\frac {\overline{y}}{|y|^{2}}-x_{2}|}}{|\frac{\overline{y}}{|y|^{2}} -x_{2}|} \biggr]f \biggl(\frac{\overline{y}}{|y|^{2}} \biggr)\frac {1}{|y|^{6}}\,dv_{y} \\& \hphantom{(2)\quad}\qquad{} -\frac{1}{4\pi} \int_{B} \biggl[\frac {e^{i\alpha|\frac{\overline{y}}{|y|^{2}}-x_{1}|}(\frac {\overline{y}}{|y|^{2}}-x_{1})}{|\frac{\overline{y}}{|y|^{2}} -x_{1}|^{3}}-\frac{e^{i\alpha|\frac{\overline {y}}{|y|^{2}}-x_{2}|}(\frac{\overline{y}}{|y|^{2}}- x_{2})}{|\frac{\overline{y}}{|y|^{2}}-x_{2}|^{3}} \biggr]f \biggl(\frac{\overline{y}}{|y|^{2}} \biggr)\frac{1}{|y|^{6}}\,dv_{y} \\& \hphantom{(2)\quad}\qquad{} + \frac{i\alpha}{4\pi} \int_{B} \biggl[\frac {e^{i\alpha|\frac{\overline{y}}{|y|^{2}}-x_{1}|}(\frac {\overline{y}}{|y|^{2}}-x_{1})}{|\frac{\overline{y}}{|y|^{2}} -x_{1}|^{2}}-\frac{e^{i\alpha|\frac{\overline {y}}{|y|^{2}}-x_{2}|}(\frac{\overline{y}}{|y|^{2}}-x_{ 2})}{|\frac{\overline{y}}{|y|^{2}}-x_{2}|^{2}} \biggr]f \biggl( \frac{\overline{y}}{|y|^{2}} \biggr)\frac{1}{|y|^{6}}\,dv_{y} \\& \hphantom{(2)\quad}\quad = I_{10}+I_{11}+I_{12}. \end{aligned}$$

Firstly, we discuss $I_{10}$. We have

$$\begin{aligned} I_{10} =& -\frac{\alpha}{4\pi} \int_{B} \biggl[\frac {e^{i\alpha|\frac{\overline{y}}{|y|^{2}}-x_{1}|}}{|\frac{\overline {y}}{|y|^{2}}-x_{1}|}-\frac{e^{i\alpha|\frac{\overline {y}}{|y|^{2}}-x_{2}|}}{|\frac{\overline{y}}{|y|^{2}}-x_{2}|} \biggr]f \biggl(\frac{\overline{y}}{|y|^{2}} \biggr)\frac {1}{|y|^{6}}\,dv_{y} \\ =& -\frac{\alpha}{4\pi} \int_{B}\frac{e^{i\alpha |\frac{\overline{y}}{|y|^{2}}-x_{1}|}-e^{i\alpha|\frac{\overline {y}}{|y|^{2}}-x_{2}|}}{|\frac{\overline{y}}{|y|^{2}}-x_{1}|}f \biggl(\frac{\overline{y}}{|y|^{2}} \biggr) \frac{1}{|y|^{6}}\,dv_{y} \\ &{}- \frac{\alpha}{4\pi} \int_{B}e^{i\alpha|\frac {\overline{y}}{|y|^{2}}-x_{2}|} \biggl(\frac{1}{|\frac{\overline {y}}{|y|^{2}}-x_{1}|}- \frac{1}{|\frac{\overline {y}}{|y|^{2}}-x_{2}|} \biggr)f \biggl(\frac{\overline {y}}{|y|^{2}} \biggr)\frac{1}{|y|^{6}} \,dv_{y} \\ =&I_{10}^{(1)}+I_{10}^{(2)}. \end{aligned}$$

By the Hölder inequality, we have

$$\begin{aligned} \bigl\vert I_{10}^{(1)} \bigr\vert \leq& \frac{|\alpha|}{4\pi} \int _{B}\frac{|e^{i\alpha|\frac{\overline {y}}{|y|^{2}}-x_{1}|}-e^{i\alpha|\frac{\overline {y}}{|y|^{2}}-x_{2}|}|}{|\frac{\overline{y}}{|y|^{2}}-x_{1}|} \biggl\vert f \biggl( \frac{\overline{y}}{|y|^{2}} \biggr) \biggr\vert \frac {1}{|y|^{6}}\,dv_{y} \\ \leq& \frac{|\alpha|}{4\pi} \int_{B}\frac {c|x_{1}-x_{2}|}{|\frac{\overline{y}}{|y|^{2}}-x_{ 1}|} \biggl\vert f \biggl( \frac{\overline{y}}{|y|^{2}} \biggr) \biggr\vert \frac{1}{|y|^{6}}\,dv_{y} \\ =& C_{9} \int_{B} \frac{1}{|\frac{\overline{y}}{|y|^{2}}-x_{1}|} \biggl\vert f \biggl( \frac{\overline{y}}{|y|^{2}} \biggr) \biggr\vert \frac {1}{|y|^{6}}\,dv_{y}|x_{1}-x_{2}| \\ \leq& C_{9} \biggl\{ \int_{B} \biggl[|y|^{-3} \biggl\vert f \biggl( \frac{\overline{y}}{|y|^{2}} \biggr) \biggr\vert \biggr]^{p}\,dv_{y} \biggr\} ^{\frac{1}{p}} \biggl\{ \int_{B}\frac{1}{|\frac {\overline{y}}{|y|^{2}}-x_{1}|^{q}|y|^{3q}}\,dv_{y} \biggr\} ^{\frac {1}{q}}|x_{1}-x_{2}| \\ =&C_{9}\bigl\| f^{(3)}\bigr\| _{L^{p}(B)}\bigl[O_{1}(x) \bigr]^{\frac{1}{q}}|x_{1}-x_{2}|. \end{aligned}$$

By (3.16) and (3.17), we have $O_{1}(x)\leq\max\{C_{3},C_{6}\} $. Therefore

3.22 $$ \bigl\vert I_{10}^{(1)} \bigr\vert \leq C_{10} \bigl\Vert f^{(3)} \bigr\Vert _{L^{p}(B)}|x_{1}-x_{2}|, $$

where $C_{10}=\max\{C_{9}C_{3}^{\frac{1}{q}},C_{9}C_{6}^{\frac {1}{q}}\}$.

By the Taylor series, we have $|e^{i\alpha|\frac{\overline {y}}{|y|^{2}}-x_{2}|}|= |e^{-b|\frac{\overline {y}}{|y|^{2}}-x_{2}|}|\leq\frac{1}{b|\frac{\overline{y}}{|y|^{2}}-x_{2}|}$. Therefore

3.23 $$\begin{aligned} \bigl\vert I_{10}^{(2)} \bigr\vert \leq& \frac{|\alpha|}{4\pi} \int_{B}e^{-b|\frac {\overline{y}}{|y|^{2}}-x_{2}|} \biggl\vert \frac{1}{|\frac{\overline {y}}{|y|^{2}}-x_{1}|}- \frac{1}{|\frac{\overline {y}}{|y|^{2}}-x_{2}|} \biggr\vert \biggl\vert f \biggl(\frac{\overline {y}}{|y|^{2}} \biggr) \biggr\vert \frac{1}{|y|^{6}}\,dv_{y} \\ \leq& \frac{|\alpha|}{4\pi} \int_{B}\frac{1}{b|\frac {\overline{y}}{|y|^{2}}-x_{2}|}\frac{||\frac{\overline {y}}{|y|^{2}}-x_{2}|-|\frac{\overline{y}}{|y|^{2}}-x_{1}||}{|\frac {\overline{y}}{|y|^{2}}-x_{1}||\frac{\overline {y}}{|y|^{2}}-x_{2}|} \biggl\vert f \biggl(\frac{\overline {y}}{|y|^{2}} \biggr) \biggr\vert \frac{1}{|y|^{6}} \,dv_{y} \\ \leq& C_{11} \int_{B}\frac{|x_{1}-x_{2}|}{|\frac {\overline{y}}{|y|^{2}}-x_{1}||\frac{\overline {y}}{|y|^{2}}-x_{2}|^{2}} \biggl\vert f \biggl( \frac{\overline {y}}{|y|^{2}} \biggr) \biggr\vert \frac{1}{|y|^{6}}\,dv_{y} \\ =& C_{11} \int_{B}\frac{1}{|\frac{\overline {y}}{|y|^{2}}-x_{1}||\frac{\overline {y}}{|y|^{2}}-x_{2}|^{2}|y|^{3}} \biggl\vert f \biggl( \frac{\overline {y}}{|y|^{2}} \biggr) \biggr\vert |y|^{-3}\,dv_{y}|x_{1}-x_{2}| \\ \leq& C_{11} \bigl\Vert f^{(3)} \bigr\Vert _{L^{p}(B)} \biggl\{ \int_{B}\frac {1}{|\frac{\overline{y}}{|y|^{2}}-x_{1}|^{q}|\frac{\overline {y}}{|y|^{2}}-x_{2}|^{2q}|y|^{3q}}\,dv_{y} \biggr\} ^{\frac {1}{q}}|x_{1}-x_{2}| \\ =&C_{11} \bigl\Vert f^{(3)} \bigr\Vert _{L^{p}(B)} \bigl[O_{3}(x)\bigr]^{\frac{1}{q}}|x_{1}-x_{2}|. \end{aligned}$$

Since

$$\begin{aligned} \biggl\vert \frac{\overline{y}}{|y|^{2}}-x_{1} \biggr\vert ^{-q} \biggl\vert \frac {\overline{y}}{|y|^{2}}-x_{2} \biggr\vert ^{-2q}|y|^{-3q} =& |y|^{-q} \biggl\vert \frac{\overline {y}}{|y|^{2}}-x_{1} \biggr\vert ^{-q}|y|^{-2q} \biggl\vert \frac{\overline {y}}{|y|^{2}}-x_{2} \biggr\vert ^{-2q} \\ \leq& C_{12} \biggl\vert y \biggl(\frac{\overline {y}}{|y|^{2}}-x_{1} \biggr) \biggr\vert ^{-q} \biggl\vert y \biggl( \frac{\overline {y}}{|y|^{2}}-x_{2} \biggr) \biggr\vert ^{-2q} \\ =&C_{12}|1-y x_{1}|^{-q}|1-y x_{2}|^{-2q}, \end{aligned}$$

we have

$$O_{3}(x)\leq C_{12} \int_{B}\frac{1}{|1-y x_{1}|^{q}|1-y x_{2}|^{2q}}\,dv_{y}=C_{12}O_{4}(x). $$

By (3.23), we have

3.24 $$ \bigl\vert I_{10}^{(2)} \bigr\vert \leq C_{13} \bigl\Vert f^{(3)} \bigr\Vert _{L^{p}(B)} \bigl[O_{4}(x)\bigr]^{\frac {1}{q}}|x_{1}-x_{2}|. $$

In the following, we discuss $O_{4}(x)$ in four cases.

(i) When $|x_{1}|\leq\frac{1}{2}$, $|x_{2}|\leq\frac{1}{2}$, as $|y|\leq1$, we have $|1-yx_{1}|\geq\frac{1}{2}$, $|1-yx_{2}|\geq \frac{1}{2}$, $|x_{1}-x_{2}|\leq1$. Hence

$$O_{4}(x)\leq \int_{B}2^{q}2^{2q}\,dv_{y}=2^{3q} \int _{B}\,dv_{y}=C_{14}. $$

As $|x_{1}-x_{2}|\leq1$, $0<\beta=\frac{p-3}{p}<1$, we have $|x_{1}-x_{2}|\leq|x_{1}-x_{2}|^{\beta}$. Therefore, by (3.24), we have

3.25 $$ \bigl\vert I_{10}^{(2)} \bigr\vert \leq C_{15} \bigl\Vert f^{(3)} \bigr\Vert _{L^{p}(B)}|x_{1}-x_{2}|^{\beta}. $$

(ii) When $|x_{1}|\geq\frac{1}{2}$, $|x_{2}|\leq\frac{1}{2}$, we have $|1-yx_{2}|\geq\frac{1}{2}$, $\frac{1}{|x_{1}|}\leq2$, $\frac {|x_{2}|}{|x_{1}|}\leq1$. Thus

$$\begin{aligned} O_{4}(x) \leq& 2^{2q} \int_{B}\frac {1}{|1-yx_{1}|^{q}}\,dv_{y}= 2^{2q}|x_{1}|^{-q} \int _{B}\frac{1}{|1-yx_{1}|^{q}|\frac{\overline {x}_{1}}{|x_{1}|^{2}}|^{q}}\,dv_{y} \\ \leq& C_{16}2^{2q}|x_{1}|^{-q} \int_{B}\frac {1}{|(1-yx_{1})\frac{\overline{x}_{1}}{|x_{ 1}|^{2}}|^{q}}\,dv_{y}= C_{16} 2^{2q}|x_{1}|^{-q} \int _{B}\frac{1}{|y-\frac{\overline{x}_{1}}{|x_{ 1}|^{2}}|^{q}}\,dv_{y}. \end{aligned}$$

Again, since

$$\begin{aligned} \frac{1}{|x_{1}|} =& \frac {1}{|x_{1}|^{\beta}} \biggl\vert \frac{\overline {x}_{1}}{|x_{1}|^{2}} \biggr\vert ^{1-\beta} = \frac{1}{|x_{1}|^{\beta}} \biggl\vert \frac{\overline {x}_{1}(x_{1}-x_{2})(\overline{x}_{1}-\overline {x}_{2})}{|x_{1}|^{2}|x_{1}-x_{2}|^{2}} \biggr\vert ^{1-\beta} \\ \leq& C_{17}\frac{1}{|x_{1}|^{\beta}} \biggl\vert \frac{\overline{x}_{1}(x_{1}-x_{2})}{|x_{1}|^{2}} \biggr\vert ^{1-\beta}\frac{1}{|x_{1}-x_{2}|^{1-\beta}} = C_{17} \frac{1}{|x_{1}|^{\beta}} \biggl\vert 1- \frac{\overline{x}_{1}x_{2}}{|x_{1}|^{2}} \biggr\vert ^{1-\beta }|x_{1}-x_{2}|^{\beta-1} \\ \leq& C_{17}|x_{1}|^{-\beta} \biggl(1+ \frac {|x_{2}|}{|x_{1}|} \biggr)^{1-\beta}|x_{1}-x_{2}|^{\beta-1} \leq C_{18}|x_{1}-x_{2}|^{\beta-1}, \end{aligned}$$

we have $|x_{1}|^{-q}\leq C_{19}|x_{1}-x_{2}|^{(\beta-1)q}$. Again from the notion that $1< q<\frac{3}{2}$, we know $\int_{B}\frac {1}{|y-\frac{\overline{x}_{1}}{|x_{1}|^{2}}|^{q}}\,dv_{y}$ is bounded. Hence,we obtain

$$O_{4}(x)\leq C_{20}|x_{1}-x_{2}|^{(\beta-1)q}. $$

Therefore, by (3.24), we have

3.26 $$\begin{aligned} \bigl\vert I_{10}^{(2)} \bigr\vert \leq& C_{13} \bigl\Vert f^{(3)} \bigr\Vert _{L^{p}(B)} \bigl[C_{25}|x_{1}-x_{2}|^{(\beta-1)q} \bigr]^{\frac {1}{q}}|x_{1}-x_{2}| \\ =&C_{21} \bigl\Vert f^{(3)} \bigr\Vert _{L^{p}(B)}|x_{1}-x_{2}|^{\beta}. \end{aligned}$$

(iii) When $|x_{1}|\leq\frac{1}{2}$, $|x_{2}|\geq\frac {1}{2}$, similar to (ii), we have

3.27 $$ \bigl\vert I_{10}^{(2)} \bigr\vert \leq C_{22} \bigl\Vert f^{(3)} \bigr\Vert _{L^{p}(B)}|x_{1}-x_{2}|^{\beta}. $$

(iv) When $|x_{1}|\geq\frac{1}{2}$, $|x_{2}|\geq\frac{1}{2}$, we have $\frac{1}{|x_{1}|}\leq2$, $\frac{1}{|x_{2}|}\leq2$. Since

$$\begin{aligned}& |1-yx_{1}|^{-q} = |1-yx_{1}|^{-q}|x_{1}|^{q}|x_{1}|^{-q} =|1-yx_{1}|^{-q} \biggl\vert \frac{\overline{x}_{1}}{|x_{1}|^{2}} \biggr\vert ^{-q}|x_{1}|^{-q} \\& \hphantom{|1-yx_{1}|^{-q}}\leq C_{23} \biggl\vert (1-yx_{1}) \frac{\overline {x}_{1}}{|x_{1}|^{2}} \biggr\vert ^{-q}|x_{1}|^{-q} = C_{23} \biggl\vert y-\frac{\overline {x}_{1}}{|x_{1}|^{2}} \biggr\vert ^{-q}|x_{1}|^{-q}, \\& |1-yx_{2}|^{-2q} =|1-yx_{2}|^{-2q}|x_{2}|^{2q}|x_{2}|^{-2q} =|1-yx_{2}|^{-2q} \biggl\vert \frac{\overline{x}_{2}}{|x_{2}|^{2}} \biggr\vert ^{-2q}|x_{2}|^{-2q} \\& \hphantom{|1-yx_{2}|^{-2q}}\leq C_{24} \biggl\vert (1-yx_{2}) \frac{\overline {x}_{2}}{|x_{2}|^{2}} \biggr\vert ^{-2q}|x_{2}|^{-2q} = C_{24} \biggl\vert y-\frac{\overline {x}_{2}}{|x_{2}|^{2}} \biggr\vert ^{-2q}|x_{2}|^{-2q}. \end{aligned}$$

We have

$$O_{4}(x)\leq C_{25} \int_{B}\frac{1}{|y-\frac{\overline {x}_{1}}{|x_{1}|^{2}}|^{q}|y-\frac{\overline {x}_{2}}{|x_{2}|^{2}}|^{2q}}\,dv_{y}. $$

Suppose $\alpha'=q$, $\beta'=2q$. Then $0<\alpha'<3$, $0<\beta '<3$, $\alpha'+\beta'=3q>3$. Thus, by Lemma 2.2, we have

$$\begin{aligned} O_{4}(x) \leq& C_{26} \biggl\vert \frac{\overline {x}_{1}}{|x_{1}|^{2}}- \frac{\overline{x}_{2}}{|x_{2}|^{2}} \biggr\vert ^{3-3q} = C_{26} \biggl\vert \frac{\overline {x}_{1}|x_{2}|^{2}-\overline {x}_{2}|x_{1}|^{2}}{|x_{1}|^{2}|x_{2}|^{2}} \biggr\vert ^{3-3q} \\ =& C_{26} \biggl\vert \frac{\overline {x}_{1}|x_{2}|^{2}-\overline{x}_{2}|x_{2}|^{2}+\overline {x}_{2}|x_{2}|^{2}-\overline {x}_{2}|x_{1}|^{2}}{|x_{1}|^{2}|x_{2}|^{2}} \biggr\vert ^{3-3q} \\ =& C_{26} \biggl\vert \frac{\overline{x}_{1}-\overline {x}_{2}}{|x_{1}|^{2}}+\frac{\overline {x}_{2}(|x_{2}|^{2}-|x_{1}|^{2})}{|x_{1}|^{2}|x_{2}|^{2}} \biggr\vert ^{3-3q} \\ \leq& C_{26} \biggl(\frac{1}{|x_{1}|^{2}}+\frac {|x_{1}|+|x_{2}|}{|x_{1}|^{2}|x_{2}|} \biggr)^{3-3q}|x_{1}-x_{2}|^{3-3q} \\ =& C_{26} \biggl(\frac{1}{|x_{1}|^{2}}+\frac {1}{|x_{1}|^{2}}+ \frac{1}{|x_{1}||x_{2}|} \biggr)^{3-3q}|x_{1}-x_{2}|^{3-3q} \\ \leq& C_{27}|x_{1}-x_{2}|^{3-3q}. \end{aligned}$$

Therefore, by (3.24), we have

3.28 $$\begin{aligned} \bigl\vert I_{10}^{(2)} \bigr\vert \leq& C_{13} \bigl\Vert f^{(3)} \bigr\Vert _{L^{p}(B)} \bigl[C_{27}|x_{1}-x_{2}|^{3-3q} \bigr]^{\frac{1}{q}}|x_{1}-x_{2}| \\ =& C_{28} \bigl\Vert f^{(3)} \bigr\Vert _{L^{p}(B)}|x_{1}-x_{2}|^{\beta}, \end{aligned}$$

where $0<\beta=\frac{p-3}{p}<1$. From (3.25)-(3.28), we obtain

3.29 $$ \bigl\vert I_{10}^{(2)} \bigr\vert \leq M_{6}^{(1)}(p) \bigl\Vert f^{(3)} \bigr\Vert _{L^{p}(B)}|x_{1}-x_{2}|^{\beta}, $$

where $M_{6}^{(1)}(p)=\max\{C_{15},C_{21},C_{22},C_{28}\}$.

By (3.22), (3.29), we obtain

3.30 $$ |I_{10}|\leq C_{10} \bigl\Vert f^{(3)} \bigr\Vert _{L^{p}(B)}|x_{1}-x_{2}|+M_{6}^{(1)}(p) \bigl\Vert f^{(3)} \bigr\Vert _{L^{p}(B)}|x_{1}-x_{2}|^{\beta}. $$

Secondly, we discuss $I_{11}$. We have

$$\begin{aligned} I_{11} =& -\frac{1}{4\pi} \int_{B} \biggl[ \frac{e^{i\alpha|\frac{\overline{y}}{|y|^{2}}-x_{1}|}( \frac{\overline{y}}{|y|^{2}}-x_{1})}{|\frac{\overline {y}}{|y|^{2}}-x_{1}|^{3}}-\frac{e^{i\alpha|\frac {\overline{y}}{|y|^{2}}-x_{2}|}(\frac{\overline{y}}{|y|^{2}} -x_{2})}{|\frac{\overline{y}}{|y|^{2}}-x_{2}|^{3}} \biggr] f \biggl(\frac{\overline{y}}{|y|^{2}} \biggr)\frac{1}{|y|^{6}}\,dv_{ y} \\ =& -\frac{1}{4\pi} \int_{B}\frac{ (e^{i\alpha |\frac{\overline{y}}{|y|^{2}}-x_{1}|}-e^{i\alpha|\frac{\overline {y}}{|y|^{2}}-x_{2}|} )(\frac{\overline {y}}{|y|^{2}}-x_{1})}{|\frac{\overline{y}}{|y|^{2}}-x_{1}|^{3}}f \biggl(\frac{\overline{y}}{|y|^{2}} \biggr) \frac{1}{|y|^{6}}\,dv_{y} \\ &{} -\frac{1}{4\pi} \int_{B}e^{i\alpha|\frac{\overline {y}}{|y|^{2}}-x_{2}|} \biggl(\frac{\frac{\overline {y}}{|y|^{2}}-x_{1}}{|\frac{\overline{y}}{|y|^{2}}-x_{1}|^{3}}- \frac {\frac{\overline{y}}{|y|^{2}}-x_{2}}{|\frac{\overline {y}}{|y|^{2}}-x_{2}|^{3}} \biggr)f \biggl(\frac{\overline {y}}{|y|^{2}} \biggr)\frac{1}{|y|^{6}} \,dv_{y} \\ =&I_{11}^{(1)}+I_{11}^{(2)}. \end{aligned}$$

Similar to $I_{10}^{(1)}$, we get

3.31 $$ \bigl\vert I_{11}^{(1)} \bigr\vert \leq C_{29} \bigl\Vert f^{(3)} \bigr\Vert _{L^{p}(B)}|x_{1}-x_{2}|. $$

By the Hölder inequality and the Hile lemma, we have

3.32 $$\begin{aligned} \bigl\vert I_{11}^{(2)} \bigr\vert \leq& \frac{1}{4\pi} \int_{B}e^{-b|\frac {\overline{y}}{|y|^{2}}-x_{2}|} \biggl\vert \frac{\frac{\overline {y}}{|y|^{2}}-x_{1}}{|\frac{\overline{y}}{|y|^{2}}-x_{ 1}|^{3}}- \frac{\frac{\overline{y}}{|y|^{2}}-x_{ 2}}{|\frac{\overline{y}}{|y|^{2}}-x_{2}|^{3}} \biggr\vert \biggl\vert f \biggl(\frac{\overline{y}}{|y|^{2}} \biggr) \biggr\vert \frac {1}{|y|^{6}}\,dv_{y} \\ \leq& C_{30} \int_{B}\frac{|\frac{\overline {y}}{|y|^{2}}-x_{1}|+|\frac{\overline{y}}{|y|^{2}}-x_{2}|}{|\frac {\overline{y}}{|y|^{2}}-x_{1}|^{2}|\frac{\overline {y}}{|y|^{2}}-x_{2}|^{2}} \biggl\vert f \biggl( \frac{\overline {y}}{|y|^{2}} \biggr) \biggr\vert \frac{1}{|y|^{6}}\,dv_{y}|x_{1}-x_{2}| \\ =& C_{30} \int_{B}\frac{1}{|\frac{\overline {y}}{|y|^{2}}-x_{1}||\frac{\overline{y}}{|y|^{2}}- x_{2}|^{2}} \biggl\vert f \biggl( \frac{\overline{y}}{|y|^{2}} \biggr) \biggr\vert \frac{1}{|y|^{6}}\,dv_{y}|x_{1}-x_{2}| \\ &{}+ C_{30} \int_{B}\frac{1}{|\frac{\overline {y}}{|y|^{2}}-x_{1}|^{2}|\frac{\overline{y}}{|y|^{2}}-x_{ 2}|} \biggl\vert f \biggl( \frac{\overline{y}}{|y|^{2}} \biggr) \biggr\vert \frac {1}{|y|^{6}}\,dv_{y}|x_{1}-x_{2}| \\ \leq& C_{31} \bigl\Vert f^{(3)} \bigr\Vert _{L^{p}(B)} \biggl[ \int_{B}\frac {1}{|1-yx_{1}|^{q}|1-yx_{2}|^{2q}}\,dv_{y} \biggr]^{\frac {1}{q}}|x_{1}-x_{2}| \\ &{} + C_{32} \bigl\Vert f^{(3)} \bigr\Vert _{L^{p}(B)} \biggl[ \int_{B}\frac {1}{|1-yx_{1}|^{2q}|1-yx_{2}|^{q}}\,dv_{y} \biggr]^{\frac {1}{q}}|x_{1}-x_{2}| \\ =&C_{31} \bigl\Vert f^{(3)} \bigr\Vert _{L^{p}(B)} \bigl[O_{4}(x)\bigr]^{\frac {1}{q}}|x_{1}-x_{2}|+C_{32} \bigl\Vert f^{(3)} \bigr\Vert _{L^{ p}(B)}\bigl[O_{5}(x) \bigr]^{\frac{1}{q}}|x_{1}-x_{2}|. \end{aligned}$$

This is similar to $I_{10}^{(2)}$ and it is easy to prove the following:

$$\begin{aligned}& C_{31} \bigl\Vert f^{(3)} \bigr\Vert _{L^{p}(B)} \bigl[{O_{4}(x)}\bigr]^{\frac {1}{q}}|x_{1}-x_{2}| \leq C_{33} \bigl\Vert f^{(3)} \bigr\Vert _{L^{p}(B)}|x_{1}-x_{2}|^{\beta}, \\& C_{32} \bigl\Vert f^{(3)} \bigr\Vert _{L^{p}(B)} \bigl[{O_{5}(x)}\bigr]^{\frac {1}{q}}|x_{1}-x_{2}| \leq C_{34} \bigl\Vert f^{(3)} \bigr\Vert _{L^{p}(B)}|x_{1}-x_{2}|^{\beta}. \end{aligned}$$

Therefore, we obtain

3.33 $$ \bigl\vert I_{11}^{(2)} \bigr\vert \leq C_{35} \bigl\Vert f^{(3)} \bigr\Vert _{L^{p}(B)}|x_{1}-x_{2}|^{\beta}. $$

By (3.31) and (3.33), we have

3.34 $$ |I_{11}|\leq C_{29} \bigl\Vert f^{(3)} \bigr\Vert _{L^{p}(B)}|x_{1}-x_{2}|+C_{35} \bigl\Vert f^{(3)} \bigr\Vert _{L^{p}(B)}|x_{1}-x_{2}|^{\beta}. $$

Finally, we discuss $I_{12}$. We have

$$\begin{aligned} I_{12} =& \frac{i\alpha}{4\pi} \int_{B} \biggl[ \frac{e^{i\alpha|\frac{\overline{y}}{|y|^{2}}-x_{ 1}|}(\frac{\overline{y}}{|y|^{2}}-x_{1})}{|\frac{\overline {y}}{|y|^{2}}-x_{1}|^{2}}-\frac{e^{i\alpha|\frac {\overline{y}}{|y|^{2}}-x_{2}|}(\frac{\overline{y}}{|y|^{2}} -x_{2})}{|\frac{\overline{y}}{|y|^{2}}-x_{2}|^{2}} \biggr]f \biggl(\frac{\overline{y}}{|y|^{2}} \biggr)\frac{1}{|y|^{6}}\,dv_{ y} \\ =& \frac{i\alpha}{4\pi} \int_{B}\frac{ (e^{i\alpha|\frac{\overline{y}}{|y|^{2}}-x_{1}|}-e^{i\alpha|\frac {\overline{y}}{|y|^{2}}-x_{2}|} )(\frac{\overline {y}}{|y|^{2}}-x_{1})}{|\frac{\overline{y}}{|y|^{2}}-x_{1}|^{2}}f \biggl(\frac{\overline{y}}{|y|^{2}} \biggr) \frac{1}{|y|^{6}}\,dv_{y} \\ &{}+ \frac{i\alpha}{4\pi} \int_{B}e^{i\alpha|\frac {\overline{y}}{|y|^{2}}-x_{2}|} \biggl(\frac{\frac{\overline {y}}{|y|^{2}}-x_{1}}{|\frac{\overline{y}}{|y|^{2}}-x_{1}|^{2}}- \frac {\frac{\overline{y}}{|y|^{2}}-x_{2}}{|\frac{\overline {y}}{|y|^{2}}-x_{2}|^{2}} \biggr)f \biggl(\frac{\overline {y}}{|y|^{2}} \biggr)\frac{1}{|y|^{6}} \,dv_{y} \\ =&I_{12}^{(1)}+I_{12}^{(2)}. \end{aligned}$$

Similar to $I_{10}^{(1)}$, we get

3.35 $$ \bigl\vert I_{12}^{(1)} \bigr\vert \leq C_{36} \bigl\Vert f^{(3)} \bigr\Vert _{L^{p}(B)}|x_{1}-x_{2}|. $$

By the Hile lemma and the Hölder inequality, we have

$$\begin{aligned} \bigl\vert I_{12}^{(2)} \bigr\vert \leq& \frac{|\alpha|}{4\pi} \int_{B}e^{-b|\frac {\overline{y}}{|y|^{2}}-x_{2}|} \biggl\vert \frac{\frac{\overline {y}}{|y|^{2}}-x_{1}}{|\frac{\overline{y}}{|y|^{2}}-x_{1}|^{2}}- \frac {\frac{\overline{y}}{|y|^{2}}-x_{2}}{|\frac{\overline {y}}{|y|^{2}}-x_{2}|^{2}} \biggr\vert \biggl\vert f \biggl(\frac{\overline {y}}{|y|^{2}} \biggr) \biggr\vert \frac{1}{|y|^{6}}\,dv_{y} \\ \leq& C_{37} \int_{B}\frac{|x_{1}-x_{2}|}{|\frac {\overline{y}}{|y|^{2}}-x_{1}||\frac{\overline {y}}{|y|^{2}}-x_{2}|^{2}} \biggl\vert f \biggl( \frac{\overline {y}}{|y|^{2}} \biggr) \biggr\vert \frac{1}{|y|^{6}}\,dv_{y} \\ =& C_{37} \int_{B}\frac{1}{|\frac{\overline {y}}{|y|^{2}}-x_{1}||\frac{\overline {y}}{|y|^{2}}-x_{2}|^{2}|y|^{3}}|y|^{-3} \biggl\vert f \biggl(\frac{\overline {y}}{|y|^{2}} \biggr) \biggr\vert \,dv_{y}|x_{1}-x_{2}| \\ \leq& C_{37} \bigl\Vert f^{(3)} \bigr\Vert _{L^{p}(B)} \biggl[ \int_{B}\frac {1}{|\frac{\overline{y}}{|y|^{2}}-x_{1}|^{q}|\frac{\overline {y}}{|y|^{2}}-x_{2}|^{2q}|y|^{3q}}\,dv_{y} \biggr]^{\frac {1}{q}}|x_{1}-x_{2}| \\ =&C_{37} \bigl\Vert f^{(3)} \bigr\Vert _{L^{p}(B)} \bigl[O_{3}(x)\bigr]^{\frac{1}{q}}|x_{1}-x_{2}|. \end{aligned}$$

Therefore

3.36 $$ \bigl\vert I_{12}^{(2)} \bigr\vert \leq C_{38} \bigl\Vert f^{(3)} \bigr\Vert _{L^{p}(B)}|x_{1}-x_{2}|^{\beta}, $$

by (3.35) and (3.36), so we have

3.37 $$ |I_{12}|\leq C_{36} \bigl\Vert f^{(3)} \bigr\Vert _{L^{p}(B)}|x_{1}-x_{2}|+C_{38} \bigl\Vert f^{(3)} \bigr\Vert _{L^{p}(B)}|x_{1}-x_{2}|^{\beta}. $$

By (3.30), (3.34), and (3.37), we have

$$\begin{aligned}& \bigl\vert \bigl(T_{\psi,\alpha}^{(2)}[f]\bigr) (x_{2})- \bigl(T_{\psi,\alpha }^{(2)}[f]\bigr) (x_{1}) \bigr\vert \\& \quad \leq M_{5}(p) \bigl\Vert f^{(3)} \bigr\Vert _{L^{p}( B)}|x_{1}-x_{2}|+ M_{6}(p) \bigl\Vert f^{(3)} \bigr\Vert _{L^{p}( B)}|x_{1}-x_{2}|^{\beta}, \end{aligned}$$

where $M_{5}(p)=C_{10}+C_{29}+C_{36}$, $M_{6}(p)=M_{6}^{(1) }(p)+C_{35}+C_{38}$.

(3) This case is similar to Theorem 3.1, and it is easy to prove. □

### Remark 3.1

Assume *B* to be as stated above and $\alpha=a+ib, b>0$. If $f\in L^{p,3}(B,\mathbb{H}(\mathbb{C}))$, $3< p<+\infty$, then

1. $|(T_{\psi,\alpha}[f])(x)|\leq M_{7}(p)\|f\| _{p,3}$, $x\in R^{3}$,
2. $|(T_{\psi,\alpha}[f])(x_{1})-(T_{\psi,\alpha }[f])(x_{2})|\leq M_{8}(p)\|f\|_{p,3}|x_{1}-x_{2}|+ M_{9}(p)\|f\|_{p,3}|x_{1}-x_{2}|^{\beta}$, $x_{1},x_{2}\in R^{3}$,
3. ${{}^{\psi}D}_{\alpha}(T_{\psi,\alpha }[f])(x)=f(x)$, $x\in R^{3}\backslash\partial B$,

where $0<\beta=\frac{p-3}{p}<1$.

## Integral representation of solution of Riemann boundary problem to inhomogeneous partial differential system

In this section, we will discuss the inhomogeneous partial differential system of first order equations as follows:

4.1 $$ \textstyle\begin{cases} \alpha w_{0}-w_{1_{x_{1}}}-w_{2_{x_{2}}}-w_{3_{x_{3}}}=c_{0}(x), \\ \alpha w_{1}+w_{0_{x_{1}}}-w_{2_{x_{3}}}+w_{3_{x_{2}}}=c_{1}(x),\\ \alpha w_{2}+w_{0_{x_{2}}}+w_{1_{x_{3}}}-w_{3_{x_{1}}}=c_{2}(x),\\ \alpha w_{3}+w_{0_{x_{3}}}-w_{1_{x_{2}}}+w_{2_{x_{1}}}=c_{3}(x), \end{cases} $$

where $w_{j}(x)$, $c_{j}(x)$ ($j=0,1,2,3$) are real-value functions.

### Problem P

Let $B\subset R^{3}$ be as stated above. The Riemann boundary value problem for system (4.1) is to find a solution $w(x)$ of (4.1) that satisfies the boundary condition

$$w^{+}(\tau)=w^{-}(\tau)G+f(\tau),\quad \tau\in\partial B, $$

where $w^{\pm}(\tau)=\lim_{x\in B^{\pm}, x\rightarrow\tau}w(x)$, $B^{+}=B$, $B^{-}=R^{3}\backslash\overline{B}$, *G* is a quaternion constant, $G^{-1}$ exists, and $f\in H^{\nu}_{\partial B}$ ($0<\nu<1$).

In fact,

4.2 $$\begin{aligned} {{}^{\psi}D}_{\alpha}[w] =& \sum_{j=1}^{3}i_{j} \frac {\partial w}{\partial x_{j}}+\alpha w \\ =& \sum_{j=1}^{3}\biggl(i_{j}i_{0} \frac{\partial w_{ 0}}{\partial x_{j}}+i_{j}i_{1}\frac{\partial w_{1}}{\partial x_{j}}+i_{j}i_{2} \frac{\partial w_{2}}{\partial x_{j}}+i_{ j}i_{3}\frac{\partial w_{3}}{\partial x_{j}}\biggr)+ \alpha\sum _{k=0}^{3}w_{k}i_{k} \\ =&(\alpha w_{0}-w_{1_{x_{1}}}-w_{2_{x_{2}}}-w_{ 3_{x_{3}}})i_{0}+( \alpha w_{1}+w_{0_{x_{1}}}-w_{2_{x_{ 3}}}+w_{3_{x_{2}}})i_{1} \\ &{}+(\alpha w_{2}+w_{0_{x_{2}}}+w_{1_{x_{3}}}-w_{ 3_{x_{1}}})i_{2}+( \alpha w_{3}+w_{0_{x_{3}}}-w_{ 1_{x_{2}}}+w_{2_{x_{1}}})i_{3}. \end{aligned}$$

Let

4.3 $$ g(x)=c_{0}(x)i_{0}+c_{1}(x)i_{1}+c_{2}(x)i_{2}+c_{3}(x)i_{3}= \sum_{j=0}^{3}c_{j}(x)i_{j}. $$

By (4.2) and (4.3), the inhomogeneous partial differential system (4.1) can be transformed to the following equation:

4.4 $$ {{}^{\psi}D}_{\alpha}[w]= \sum_{j=0}^{3}c_{j}(x)i_{j}=g(x). $$

Therefore Problem P as stated above can be transformed to Problem Q.

### Problem Q

Let $B\subset R^{3}$ be as stated above. The Riemann boundary value problem for system (4.1) is to find a solution $w(x)$ of (4.4) that satisfies the boundary condition

$$w^{+}(\tau)=w^{-}(\tau)G+f(\tau), \quad \tau\in\partial B, $$

where $w^{\pm}(\tau)=\lim_{x\in B^{\pm}, x\rightarrow\tau}w(x)$, $B^{+}=B$, $B^{-}=R^{3}\backslash\overline{B}$, *G* is a quaternion constant, $G^{-1}$ exists, and $f\in H^{\nu}_{\partial B}$ ($0<\nu<1$).

### Theorem 4.1

*Let* *B* *be as stated above*. *Find a quaternion*-*valued function* $u(x)$ *satisfying the system* ${{}^{\psi}D}_{\alpha}[u]=0(x\in R^{3}\backslash \partial B)$ *and vanishing at infinity with the boundary condition*

4.5 $$ u^{+}(\tau)=u^{-}(\tau)G+f(\tau), \quad \tau\in\partial B, $$

*where* $u^{\pm}(\tau)=\lim_{x\in B^{\pm}, x\rightarrow\tau}u(x)$, *G* *is a quaternion constant*, $G^{-1}$ *exists*, *and* $f\in H^{\lambda }_{\partial B}$ ($0<\lambda<1$). *Then the solution can be expressed as*

$$u(x)= \textstyle\begin{cases} \int_{\partial B}\mathcal{K}_{\psi,\alpha }(y-x)\,d\sigma_{y}f(y),& x\in B^{+}, \\ \int_{\partial B}\mathcal{K}_{\psi,\alpha }(y-x)\,d\sigma_{y}f(y)G^{-1},& x\in B^{-}. \end{cases} $$

### Proof

Define

$$\varphi(x)= \textstyle\begin{cases} u(x),& x\in B^{+}, \\ u(x)G,& x\in B^{-}. \end{cases} $$

Then it is obvious that ${{}^{\psi}D}_{\alpha}[\varphi]=0$ ($x\in R^{3}\backslash\partial B$) and the Riemann boundary condition (4.5) is equivalent to

$$\varphi^{+}(\tau)=\varphi^{-}(\tau)+f(\tau),\quad \tau\in \partial B. $$

Suppose $\Psi(x)=\int_{\partial B}\mathcal{K}_{\psi,\alpha }(y-x)\, d\sigma_{y}f(y)$. Then ${{}^{\psi}D}_{\alpha}[\Psi]=0$ ($x\in R^{3}\backslash\partial B$). By the Plemelj formula, we have

$$\Psi^{+}(\tau)-\Psi^{-}(\tau)=f(\tau),\quad \tau\in \partial B. $$

Hence $\varphi^{+}(\tau)-\Psi^{+}(\tau)=\varphi^{-}(\tau)-\Psi ^{-}(\tau)$ ($\tau\in\partial B$). Thus ${{}^{\psi}D}_{\alpha}[\varphi -\Psi]=0$ and by Theorem 3.12 in [10] we obtain $\varphi(x)=\Psi(x)$. So the solution can be expressed as

$$u(x)= \textstyle\begin{cases} \int_{\partial B}\mathcal{K}_{\psi,\alpha }(y-x)\,d\sigma_{y}f(y),& x\in B^{+}, \\ \int_{\partial B}\mathcal{K}_{\psi,\alpha }(y-x)\,d\sigma_{y}f(y)G^{-1},& x\in B^{-}. \end{cases} $$

 □

### Theorem 4.2

*Let* *B* *be as stated above and* $g(x)\in L^{p,3}(R^{3},\mathbb {H}(\mathbb{C}))$, $3< p<+\infty$. *Find a quaternion*-*valued function* $w(x)$ *satisfying the system* ${{}^{\psi}D}_{\alpha}[w](x)=g(x)$ ($x\in R^{3}\backslash\partial B$) *and vanishing at infinity with the boundary condition*

4.6 $$ w^{+}(\tau)=w^{-}(\tau)G+f(\tau), \quad \tau\in\partial B, $$

*where* $w^{\pm}(\tau)=\lim_{x\in B^{\pm}, x\rightarrow\tau}w(x)$, *G* *is a quaternion constant*, $G^{-1}$ *exists*, *and* $f\in H^{\lambda }_{\partial B}$ ($0<\lambda<1$). *Then the solution has the form*

$$w(x)=\Psi(x)+\bigl(T_{\psi,\alpha}[g]\bigr) (x), $$

*in which* ${{}^{\psi}D}_{\alpha}[\Psi]=0$ *and*

$$\Psi(x)= \textstyle\begin{cases} \int_{\partial B}\mathcal{K}_{\psi,\alpha }(y-x)\,d\sigma_{y}\tilde{f}(y),& x\in B^{+}, \\ \int_{\partial B}\mathcal{K}_{\psi,\alpha }(y-x)\,d\sigma_{y}\tilde{f}(y)G^{-1},& x\in B^{-}, \end{cases} $$

*where* $\tilde{f}=f+(T_{\psi,\alpha}[g])(G-1)$.

### Proof

By Remark 3.1, we know ${{}^{\psi}D}_{\alpha}[w]={{}^{\psi}D}_{\alpha }[\Psi(x)+(T_{\psi,\alpha}[g])(x)]=g(x)$. The boundary condition (4.6) is equivalent to

4.7 $$ \bigl(\Psi+T_{\psi,\alpha}[g]\bigr)^{+}(\tau)=\bigl( \Psi+T_{\psi,\alpha }[g]\bigr)^{-}(\tau)G+f(\tau),\quad \tau\in\partial B. $$

Again from Remark 3.1, we know that $(T_{\psi,\alpha}[g])(x)$ has continuity in $R^{3}$. Thus $(T_{\psi,\alpha}[g])^{+}=(T_{\psi ,\alpha}[g])^{-}=T_{\psi,\alpha}[g]$, so (4.7) is equivalent to

4.8 $$ \Psi^{+}(\tau)=\Psi^{-}(\tau)G+\bigl(T_{\psi,\alpha}[g] \bigr) (\tau ) (G-1)+f(\tau),\quad \tau\in\partial B. $$

Suppose $\tilde{f}=f+(T_{\psi,\alpha}[g])(G-1)$. Then (4.8) has the following form:

4.9 $$ \Psi^{+}(\tau)=\Psi^{-}(\tau)G+\tilde{f}(\tau),\quad \tau \in \partial B. $$

Again from Theorem 4.1, the solutions which satisfy the system ${{}^{\psi }D_{\alpha}}[\Psi]=0$ and boundary condition (4.9) can be represented in the form

$$\Psi(x)= \textstyle\begin{cases} \int_{\partial B}\mathcal{K}_{\psi,\alpha }(y-x)\,d\sigma_{y}\tilde{f}(y),& x\in B^{+}, \\ \int_{\partial B}\mathcal{K}_{\psi,\alpha }(y-x)\,d\sigma_{y}\tilde{f}(y)G^{-1},& x\in B^{-}, \end{cases} $$

where $\tilde{f}=f+(T_{\psi,\alpha}[g])(G-1)$. □

### Remark 4.1

By Theorem 4.2, the solution of problem *P* can be expressed as

$$w(x)=\Psi(x)+\bigl(T_{\psi,\alpha}[g]\bigr) (x), $$

in which ${{}^{\psi}D}_{\alpha}[\Psi]=0$ and

$$\Psi(x)= \textstyle\begin{cases} \int_{\partial B}\mathcal{K}_{\psi,\alpha }(y-x)\,d\sigma_{y}\tilde{f}(y),& x\in B^{+}, \\ \int_{\partial B}\mathcal{K}_{\psi,\alpha }(y-x)\,d\sigma_{y}\tilde{f}(y)G^{-1},& x\in B^{-}, \end{cases} $$

where $\tilde{f}=f+(T_{\psi,\alpha}[g])(G-1)$.
